# Repeated evolution of blanched coloration in a lizard across independent white‐sand habitats

**DOI:** 10.1002/ece3.9555

**Published:** 2022-12-07

**Authors:** Telma G. Laurentino, Drew E. Dittmer, Maggie R. Grundler, Francisco Pina‐Martins, Janey Haddock, Toby J. Hibbitts, Erica Bree Rosenblum

**Affiliations:** ^1^ Department of Environmental Science, Policy, and Management University of California Berkeley California USA; ^2^ Museum of Vertebrate Zoology University of California Berkeley California USA; ^3^ Erell Institute Lawrence Kansas USA; ^4^ cE3c‐Centre for Ecology, Evolution and Environmental Changes, Faculdade de Ciências Universidade de Lisboa (ULisboa) Lisbon Portugal; ^5^ Division of Mammalogy Biodiversity Institute, University of Kansas Lawrence Kansas USA; ^6^ Biodiversity Research and Teaching Collection, Department of Ecology and Conservation Biology Texas A&M University College Station Texas USA; ^7^ Natural Resources Institute Texas A&M University College Station Texas USA

**Keywords:** convergence, evolution, *Holbrookia maculata*, lizard, MC1R, parallelism, phenotype, Salt Basin dunes, White Sands

## Abstract

The White Sands lizards of New Mexico are a rare and classic example of convergent evolution where three species have evolved blanched coloration on the white gypsum dunes. Until now, no geological replicate of the pattern had been described. However, one of the White Sands species, the lesser earless lizard (*Holbrookia maculata*), has been discovered to also inhabit the Salt Basin Dunes of Texas, where it has also evolved a blanched morph. We here present a first phenotypic and genetic description of the Salt Basin Dunes population of *H. maculata*. Phylogenetic inference based on a housekeeping gene (ND4) and a classic candidate gene in the melanin‐synthesis pathway (Melanocortin 1 Receptor; *Mc1r*) shows the newly discovered population as an independent lineage, with no evidence of genetic parallelism in the coding region of *Mc1r*. Initial morphological data suggest that while this population displays convergent evolution in blanched coloration, there are divergent patterns in limb length and habitat use behavior between the gypsum environments. Our findings present the White Sands/Salt Basin Dunes as an exceptionally promising comparative model for studies of adaptation and convergent evolution.

## Graphical abstract

The White Sands lizards of New Mexico are a rare and classic example of convergent evolution where three species have evolved blanched coloration on the white gypsum dunes. We present a geological replicate where the lesser earless lizard (*Holbrookia maculata*) has also evolved a blanched morph. We present a first phenotypic and genetic description of the Salt Basin Dunes population of *H. maculata*. The population seems to constitute and independent genetic lineage and despite convergence in dorsal coloration, we find divergence in other traits.

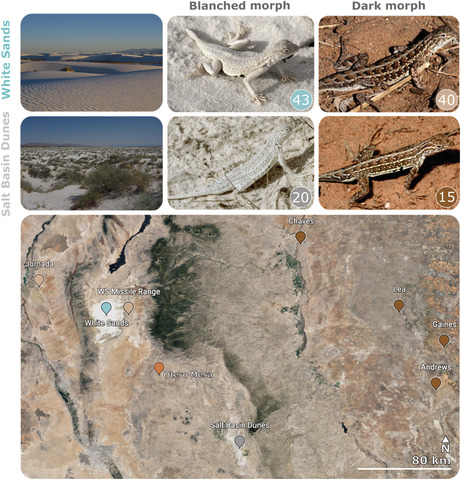

## 1 INTRODUCTION

Understanding how species adapt to environmental change remains one of the central goals of evolutionary research (Bolnick et al., 2018). The pace of contemporary global environment change lends increased importance and urgency, as data on species response to habitat shifts becomes increasingly valuable for designing effective biodiversity conservation strategies (Hendry et al., 2010; Mace & Purvis, 2008; Urban et al., 2016). Examples of repeated and convergent evolution for organisms adapting to natural and abrupt environmental transitions provide powerful opportunities to study the ecological, morphological, and genetic factors underlying adaptation (Conte et al., 2012; Gompel & Prud'homme, 2009; Rosenblum et al., 2014, 2017).

The White Sands of New Mexico is a compelling system to study convergent adaptation of independent lineages to a geologically young (~5000 years; Kocurek et al., 2007; Langford, 2003) and abrupt (over mere meters of ecotone; Des Roches et al., 2017; Rosenblum et al., 2017) habitat transition. Three lizard species ‐ *Sceloporus cowlesi, Aspidoscelis inornata* and *Holbrookia maculata* ‐ have successfully colonized and adapted to the White Sands, independently evolving blanched morphotypes. The blanched morphs are well‐matched to the white gypsum background and strikingly contrast with their brown conspecifics found across the species darker soil range (Rosenblum et al., 2004, 2007, 2010). Blanched coloration increases camouflage, mainly against avian predators (Hardwick et al., 2015), while maintaining thermal performance (Gunderson et al., 2022).

**FIGURE 1:**
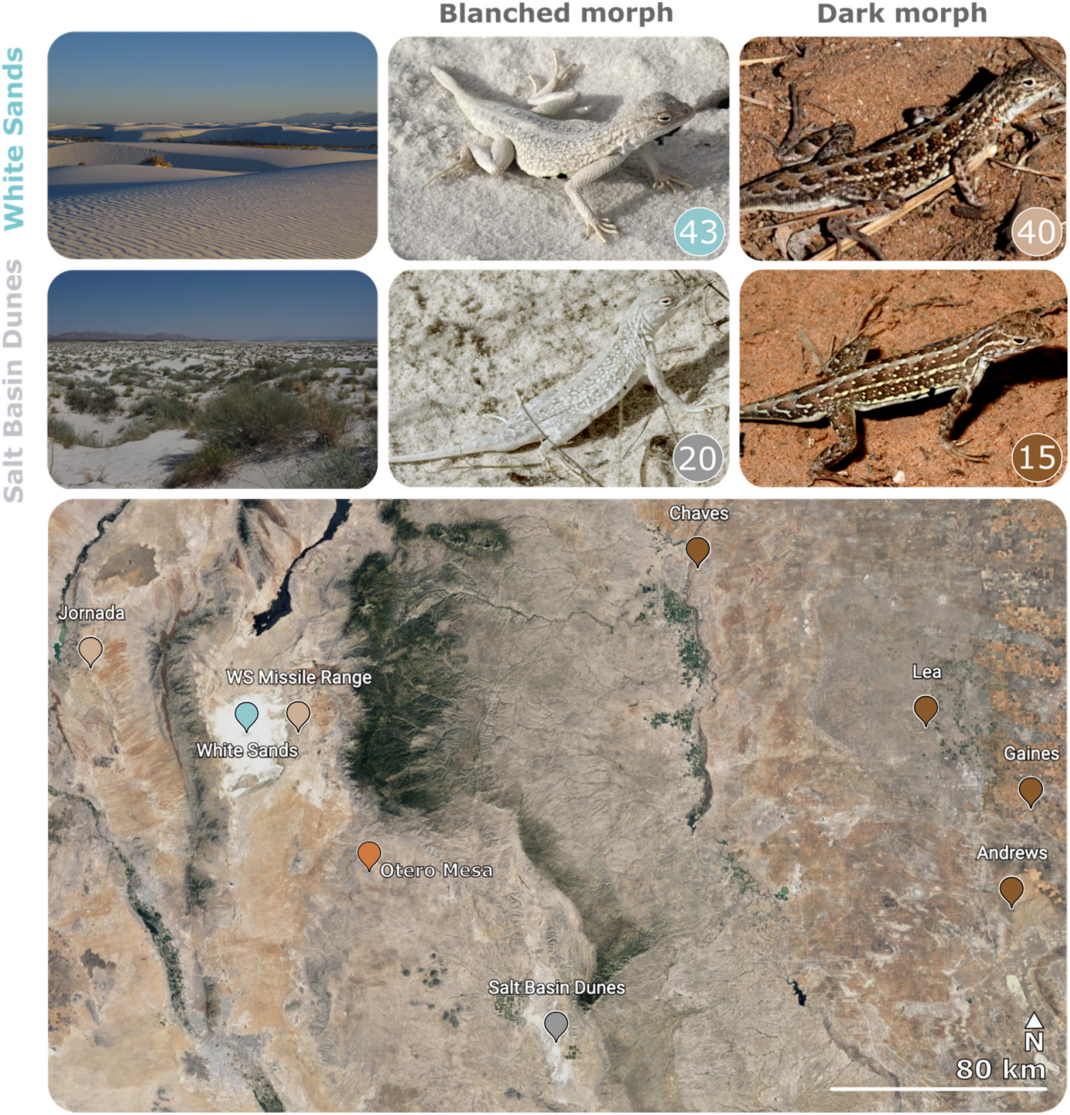
Study systems and sampling locations. Numbers in bubbles are the total number of individuals used across analysis. Population colors are consistent throughout all figures, with dark soil populations to the West (NM) depicted in light brown, dark soil populations to the East (NM and TX) depicted in darker brown and a population from a region between WS and SBD (Otero Mesa, NM) depicted in orange. Map created with Google Earth (Landsat/Copernicus). Photo credits: DED, TGL, TJH, and Alex Krohn.

Here, we describe an analogous and independent gypsum dune system where one of the species, *Holbrookia maculata*, has once again colonized a white sand habitat and evolved a strikingly blanched coloration. The Salt Basin Dunes (*SBD* from here on), in the Guadalupe Mountains National Park, Texas, have a geological origin similar to the White Sands (*WS* from here on) but are smaller (Figure 1) and less studied. Previously undescribed, the local *H. maculata* population is allopatric from the White Sands, and all known *H. maculata* outside the Salt Basin Dunes exhibit the ancestral brown dorsal coloration that camouflages in the adobe soils of the Chihuahuan Desert.

We hypothesize that the SBD *H. maculata* represent an independent colonization event with subsequent repeated evolution of blanched cryptic coloration. This species is highly patchy in its distribution (Degenhardt et al., 2005; Stebbins, 2003), and is not continuously distributed between the two habitats, which are approximately 160 km (99.4 miles) apart. Furthermore, given intense predator selection against conspicuous morphs (Robertson et al., 2011), it is highly unlikely that blanched individuals could successfully migrate across the dark soils separating both habitats.

Here, we conduct a morphological and genetic exploration of the Salt Basin Dunes blanched *H. maculata* population. We do so within an evolutionary comparative context with the White Sands blanched population and several dark soil populations for comparison, thus establishing this lizard and gypsum dune systems as an ideal model to study replicated evolution across populations of the same species.

## 2 MATERIAL AND METHODS

### 2.1 Salt Basin dunes description and comparison to the White Sands

The Salt Basin Dunes (SBD) are located in Texas, west of the Guadalupe Mountains, in the Guadalupe Mountains National Park (Figure 1). This gypsum dune system covers an area of approximately 8.03 km^2^ (3.1 square miles), and the dunes range from three‐feet high and heavily vegetated dunes at the south end of the area, to sixty‐feet high and largely non‐vegetated dunes at the north end. Also within the Chihuahuan desert, approximately 145 km (90 square miles) northwest, is the White Sands National Park of New Mexico, which is considerably larger, covering 712 km^2^ (275 square miles; Figure 1).

While the geological age of the White Sands has been determined (2000–5000 years old; Kocurek et al., 2007; Langford, 2003), the origin of the SBD remains to be dated. However, the geological and tectonic history of the region provides some cues for a similarly recent origin (<10,000 years old). The Salt Basin is a graben (tectonic valley), and the streams surrounding the graben drained into a basin with no outlet, where gypsum and salt were deposited as water evaporated. During the Pleistocene (10,000 to 1.8 million years ago), heavier rainfall and lower temperatures maintained a shallow lake that would spread over the lowest portions of the Salt Basin. Similarly to the history of the White Sands, the evaporation of the local lake and the wind erosion of the lake bed contributed to the formation of the gypsum dunes to the west (Boyd & Kreitler, 1986; Given, 2004; Lee et al., 2012; Szynkiewicz et al., 2010).

The WS and SBD also differ in their lizard communities. In addition to the blanched *H. maculata*, SBD also harbor predatory leopard lizards (*Gambelia wislizenii*), side‐blotched lizards (*Uta stansburiana*) and at least one large species of whiptails (*Aspidoscelis sp.)* within the white dune habitat. These additional species do not show obvious evidence of blanching. In the White Sands by contrast, the only species known to inhabit the dunes are the blanched trio of: *Holbrookia maculata*, *Sceloporus cowlesi* and *Aspidoscelis inornata*. The *Uta stansburiana* in the White Sands area are restricted to the dark soils surrounding the dunes.

Another noteworthy difference is the vegetation distribution, which in the White Sands is restrained to the interdunal area. Comparatively, in the Salt Basin Dunes the vegetation cover is more homogenously widespread, with the whole area having generally lower and more consistently covered dunes. In sum, the Salt Basin dunes cover a considerably smaller area (Figure 1), with higher vegetation cover and a different lizard community.

### 2.2 Lizard sampling

To situate the newly discovered SBD population in an evolutionary context, we applied a repeated pair approach including individuals from each morph (blanched and dark) across the two gypsum dune systems (Figure 1). We selected dark morph individuals from populations as close as possible to each gypsum habitat, with proximity limited by the characteristically patchy distribution of the species (Degenhardt et al., 2005; Stebbins, 2003). Blanched populations were sampled from the White Sands (WS) and Salt Basin Dunes (SBD); dark populations were sampled to the West (Jornada and White Sands Missile Range); at a region between WS and SBD (Otero Mesa) and the to the East (Andrews, Chaves, Gaines, Lea; Figure 1).

All lizards were individually collected with a lasso and handled to minimize stress.

All field research, specimen collection, and tissue collection was approved by local and state agencies, including National Park Service Scientific Research and Collecting Permits (Permit#: GUMO‐2018‐SCI‐0015; WS‐2020‐SCI‐0004; TX‐SPR‐0506‐662; GUMO‐2022‐SCI0010; NMDGF‐3353).

All detailed individual data can be found in Appendix S1.

### 2.3 Genetic analysis

The Melanocortin 1 receptor (*Mc1r*) gene is known to affect melanin synthesis across a range of vertebrates (Hoekstra, 2006; Hubbard et al., 2010; Rosenblum et al., 2004). Previous studies in the White Sands showed that coloration is genetically determined, and candidate gene approaches paired with functional assays showed that variation between blanched and dark morphs in two White Sands species (*Sceloporus cowlesi* and *Aspidoscelis inornata*) is influenced by coding mutations in the *Mc1r* gene (Rosenblum et al., 2010). Variation at *Mc1r* was also associated with coloration for *Holbrookia maculata*, but the resulting amino acid substitution had no detectable functional effect (Rosenblum et al., 2010). These results do not exclude the possibility that *Mc1r* could play a role in blanched coloration for White Sands *H. maculata* or an independent population in SBD (e.g., observed mutations may be in linkage disequilibrium with upstream noncoding mutations or may impact receptor function in ways that were not measured).

One way of assessing signatures of parallel evolution is through the phylogenetic patterns of lineage sorting between different molecular markers. If mutations at the *Mc1r* locus (that are themselves functionally relevant or linked with other causative mutations) are shared between the WS and SBD blanched populations, then we expect the *Mc1r* phylogeny to show clustering by phenotype (blanched WS closer to SBD). Contrastingly, the ND4 phylogeny should recapitulate the geographic proximity of populations (Colosimo et al., 2005; Schluter & Conte, 2009).

To infer phylogenetic patterns, genomic DNA was extracted (Qiagen DNeasy Blood & Tissue kit Cat. No.: 69504) from blanched SBD individuals (*N* = 10), from a population between WS and SBD (Otero Mesa *N* = 3), and from 11 dark individuals from four neighboring dark soil populations (Andrews *N* = 3; Chaves *N* = 4; Gaines *N* = 1; Lea *N* = 3; Figure 1). For the WS system, we retrieved GenBank data from our previous sequencing efforts for 30 blanched individuals, and 19 dark individuals from two neighboring dark populations (White Sands Missile Range *N* = 4; Jornada *N* = 15). All downloaded and newly produced sequence accession numbers are available in Appendix S1, organized per dune system, population and gene.

The SBD ND4 (797 bp) and *Mc1r* (923 bp) coding sequences were amplified following the PCR protocol previously established for the *H. maculata* of the WS (Rosenblum et al., 2004, 2010) and Sanger sequenced on an ABI3130xl instrument (Applied Biosystems). Sequence files were analyzed and exported as FASTA sequences using SeqTrace 0.9.1 (Stucky, 2012). The sequences of both genes were aligned using mafft v7.407 (Katoh & Standley, 2013), and trimmed at the 5′ end to the start codon, and on the 3′ end to the stop codon, thus keeping the coding sequences, which were used to infer the phylogenies. Maximum likelihood analyses were conducted using RAxML v 8.2.12 (Stamatakis, 2014), using the original search algorithm and the “GTRCAT” approximate model. Fast bootstrap search was used to compute branch support (1000 replicates). A seed value of 112,358 was used for calculating the initial maximum parsimony tree and for bootstrapping. Bayesian inference trees were calculated using MrBayes v3.2.6 (Ronquist et al., 2012), using the default priors and 1,500,000 generations. To root both phylogenies, we chose another lizard from the Phrynosomatidae family as an outgroup: the eastern fence lizard (*Sceloporus cowlesi)*, from which sequences for both gene regions were obtained from NCBI (Accession numbers Nd4: EU045304.1; Mc1r: AY586153.1). The inferred phylogenetic trees were plotted with a python script using the toytree v 1.0 library (Eaton, 2020). The complete analysis pipeline and respective parameters are publicly available as a gitlab repository: https://gitlab.com/StuntsPT/the‐colours‐of‐guadalupe.

**FIGURE 2:**
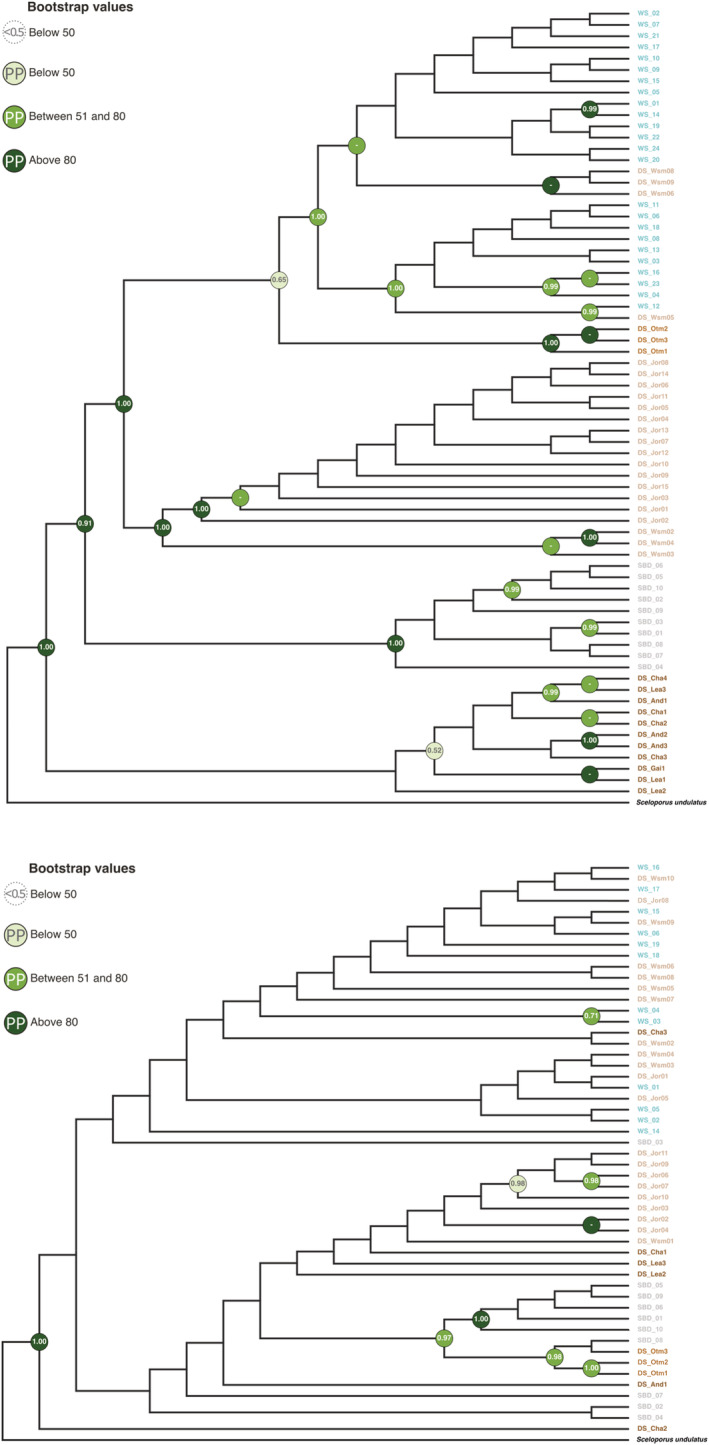
Phylogenetic trees for the mitochondrial gene (ND4, *top*) and for the melanocortin 1 receptor gene (*Mc1r*, *bottom*). The ND4 tree generally recapitulates the geographic structure among populations. The *Mc1r* tree shows less clear patterns of lineage sorting by geographic location. All SBD‐specific base‐pair genetic changes are synonymous. Circles at nodes contain information on bootstrap value (color‐coded in green scale) and posterior probabilities (number within). When the bootstrap value is below 50 and the posterior probability is below 0.5 no circle is displayed. Labels for individuals are color‐coded by environment and named by population as in Figure 1. Cladograms are shown to facilitate lineage sorting visualization, but see Figure S1, in appendix S2, for phylograms with branch length information.

Because this investigation focused on the lineage sorting patterns between gypsum dune habitats, we present the cladograms as main figures (Figure 2), which facilitate the visualization of lineage sorting, but see Supplementary figures (Figure S1, in appendix S2) for the phylograms with branch length information.

### 2.4 Color pattern analysis

To acquire dorsal coloration data, 20 individuals (10 males; 10 females) from each blanched population were photographed *in loco*, inside a portable white photo studio box (*Neewer*), together with a reference color scale (*X‐Rite ColorChecker Classic Mini*). The color scale and each lizard were placed inside an open and transparent Lee's kritter keeper, inside the photo cube (40 cm^3^). The photo cube was placed in unshaded ground, and only had a small opening at the top that fits around the camera lenses, creating a stable light environment without shades or unbalanced sun incidence on the animal. After placing the lizard within the photo cube, we recorded dorsal temperature with a laser thermometer and took a color photo with a *Canon Powershot G7X – Mark II*, framing both the lizard and the color scale, which was used to further standardize picture color across all specimens.

Stress (Seddon & Hews, 2016), temperature (Rosenblum, 2005) and ontogeny (Escudero et al., 2016; Hawlena et al., 2006) may affect dorsal brightness, thus we minimized capture stress by handling the animals as little as possible. All lizards were captured by lasso within ~3 min of first observation, placed in a mesh bag, transported into the photo cube, and processed. To minimize confounding effects of ontogeny and temperature when selecting individuals representative of the White Sands and Salt Basin Dunes dorsal variation, the 20 pairs of lizards analyzed were matched for sex, dorsal temperature at the time of the photo, and SVL, without looking at the photos (Figure S2, in appendix S2).

Color profiles of all photos were calibrated and standardized in Adobe Lightroom (*v. 5.4)* using the X‐Rite classic color scale. The phenotypic area for color analysis was defined as the dorsal area between the hind and forelimbs. Each color calibrated individual photo was cropped, and background masked in GIMP (*v. 2.10*; The GIMP Development Team, 2019). The dorsal areas were then analyzed using the R packages *Colordistance* (Weller & Westneat, 2019) to retrieve luminance data, and *Recolorize* (Weller et al., 2021) to visualize dorsal color K‐means per sex (Hager, 2001b), and per population. Differences between populations were measured through effect size estimates (Hedge's g and confidence interval) between comparison groups. All analyses were performed in R studio, R ver. 4.0.2 (R Core Team, 2019).

### 2.5 Morphometric analysis

We conducted basic morphometric analyses of the blanched SBD population compared with WS blanched *H. maculata* and East and West dark soils populations. A total of 20 blanched individuals (7 females, 12 males, 1 unassigned) were sampled de novo at the SBD (GPS 31.91656, −104.98973) in the summer of 2018 (between May 30th and June 4th). For each individual, snout to vent length (SVL) was measured with a ruler; and handheld calipers were used to measure head size: width, depth, length; and limb size: length of the right femur, and right rear toe (from heel to tip of fourth toe). The same measurements were taken from museum specimens for the remaining study populations: 19 (8 females, 11 males) blanched individuals from the White Sands; 18 from East dark soil populations (Jornada: 6 females, 3 males; White Sands missile range: 5 females, 1 males); and 15 (12 females, 3 males) from East dark soil populations.

Morphometric data for SBD, WS, and East dark soil populations were collected by the same experimenter (DED), with West dark soil being an exception (TGL). No Morphometric data was available for the three Otero Mesa individuals. Individual measurements and all raw data are available in Appendix S1.

**FIGURE 3:**
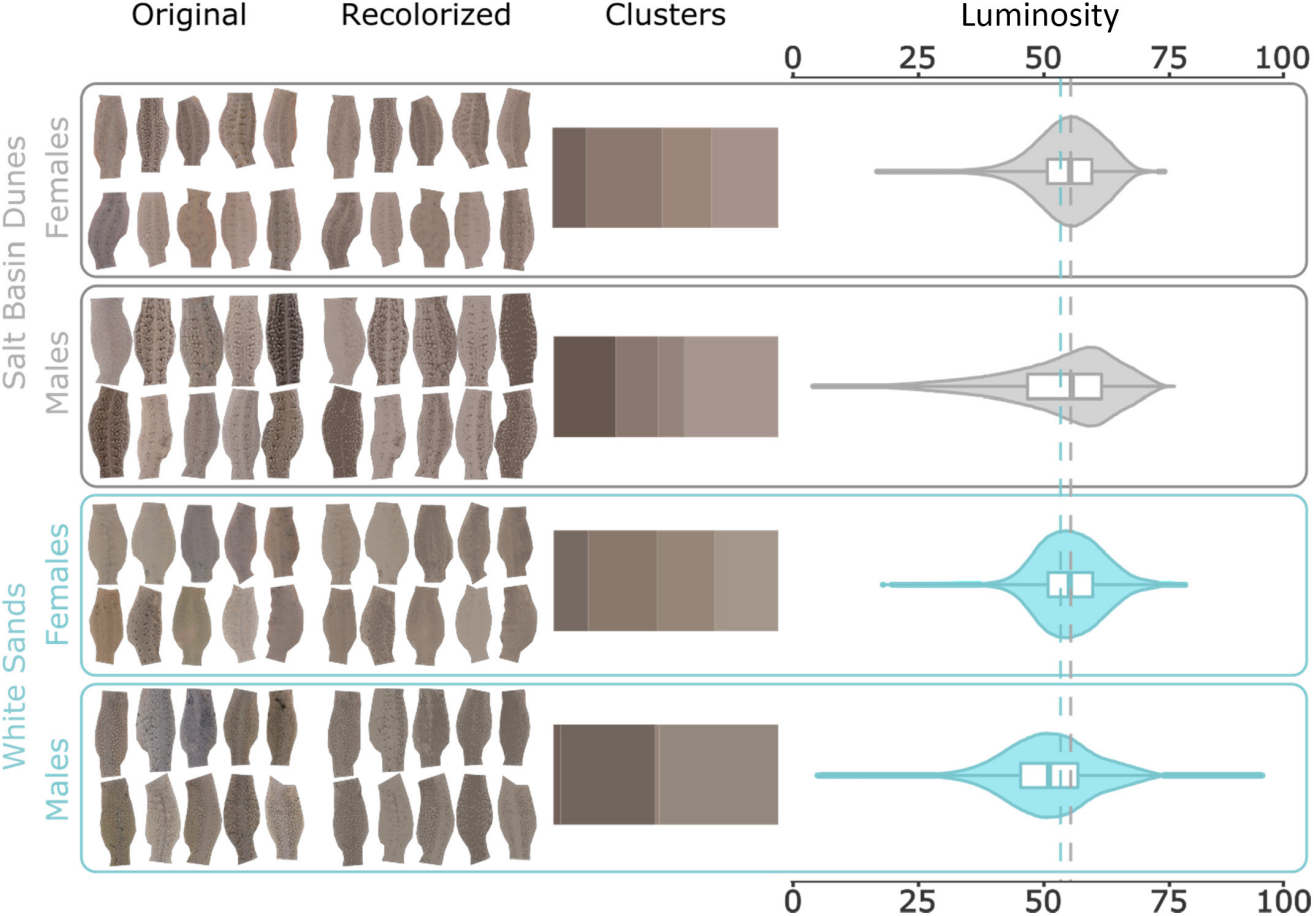
Dorsal coloration comparison between white sand populations. From left to right: original standardized photos of the dorsal area comprised between limb insertions; Recolorized images and color clusters are the result of dorsal pixels being binned into K‐means (second column: recolorized dorsal areas; third columns: population K‐means); violin and box plots of dorsal luminosity per sex, with vertical dashed lines indicating the population (males and females pooled) median (WS = 53.34; SBD = 55.33). Higher values of luminosity indicate overall lighter coloration. The difference in luminosity between populations is not statistically supported.

We looked at the relationship between body size (SVL) and both head size (Figure S3, in appendix S2) and limb length (Figure 3), traits known to vary between blanched and dark conspecifics in the White Sands system (Des Roches et al., 2014; Rosenblum & Harmon, 2011). To further visualize the morphological differences between SBD and the remaining populations, we applied a standard regression‐based approach (Berner, 2011; Hagey et al., 2017) to calculate the individual residuals of the regression of the limb length/head size against body size. To statistically quantify the uncovered differences in limb length, we applied tests of normality (Shapiro–Wilk test) and homoscedasticity (Levene's test) to the residuals of SBD and non‐SBD populations. All data proved to be normal (*p*‐values >.266) yet not homoscedastic (*p*‐values <.0254). We thus applied a non‐parametric test (Mann–Whitney‐Wilcoxon) to quantify the differences in femur and rear toe lengths between SBD and the remaining populations. All data analysis and visualization were conducted using R (*v 4.0.2*; R Core Team, 2019) in Rstudio (*v 1.3.1093*) and all code is available.

## 3 RESULTS & DISCUSSION

The White Sands system has offered an exceptional model to study repeated evolution across species (Rosenblum et al., 2010). The discovery of an additional blanched population of the lesser earless lizard (*Holbrookia maculata*) in the Salt Basin Dunes enhances the study power of a classical model by adding an independent geological replicate within species. By comparing the blanched SBD population with conspecifics from (i) the well‐studied and phenotypically convergent population from the White Sands; and (ii) with several nearby dark soil populations, we provide a first morphological and genetic exploration and raise ecomorphological hypotheses which can provide a robust basis for future studies.

### 3.1 Salt Basin dunes blanched *H. maculata* form an independent genetic lineage and there is no evidence of genetic parallelism at the Mc1r coding region

The phylogeny based on the mitochondrial ND4 gene (Figure 2, top) shows an overall clear lineage sorting pattern across populations recapitulating population proximity and potential geographic barriers to gene flow. The dark soil populations to the East (Chaves, Lea, Gaines and Andrews) are the most divergent populations, followed by the branch of the Salt Basin dunes blanched population. The Northwest population group shows Jornada as an independent branch, which is concordant with the San Andres mountain range acting as a barrier to ongoing gene flow. White Sands blanched individuals cluster with the neighboring brown individuals from the White Sands missile range with Otero Mesa as a sister branch. This biogeographic pattern is consistent with expectations of isolation by distance given the patchy distribution of the species and suggests that the SBD population may indeed be an independent evolutionary lineage.

However, the *Mc1r* gene tree (Figure 2, bottom) shows a lack of overall resolution of clusters and comparatively weaker nodal support (the majority of nodes have bootstraps values and posterior probabilities below 50). For the nuclear marker, the SBD population seems to be closer to the Otero Mesa population and the lineage sorting of the Eastern dark soil and Jornada populations is lost. It is thus difficult to conclusively infer the biogeographical history of *H. maculata* in this region without additional genetic data. Mito‐nuclear discordances are common (Toews & Brelsford, 2012): lineage sorting is often more pronounced for mitochondrial genes, which typically have higher mutation rates and one‐fourth of the effective population size (due to maternal inheritance). Thus, mitochondrial markers show stronger demographic effects of genetic drift compared with nuclear markers, which are diploid and subjected to meiotic recombination (Toews & Brelsford, 2012). It is possible that the discordant patterns between these two markers reflect changes in connectivity between populations associated with the geology of the Tularosa Basin. However, a robust interpretation will require higher resolution genomic data and a more comprehensive sampling of dark soil populations (which is hindered by land‐use change and population declines).

The blanched population from SBD does have several SNPs in the *Mc1r* sequence not found in other populations (with only one being monomorphic within SBD, Appendix S1). All of these SNPs are synonymous mutations that would not impact receptor function or differences in coloration, suggesting that coding mutations at *Mc1r* are not causative of blanched coloration in SBD *H. maculata*, unless they are linked to causative variants elsewhere in the genome. This is similar to what was found for the White Sands population (Rosenblum et al., 2004, 2010).

Overall, we found that SBD and WS populations *of H. maculata* seem to be independent genetic lineages without direct evidence for genetic parallelism in the coding region of *Mc1r*. This does not exclude the possibility of parallelism at other genomic regions and/or shared changes in *Mc1r* gene regulation that play a role in blanched coloration, as suggested before (S. Des Roches et al., 2017; Laurent et al., 2016; Rosenblum et al., 2007, 2017). Furthermore, other lizard species showing variation in melanic phenotypes often lack associated variation at *M1cr* (Buades et al., 2013; Corso et al., 2012; Nunes et al., 2011), suggesting that other genes in the melanin‐synthesis pathway are likely involved in convergent phenotypes.

The evolution of adaptive traits in novel environments can occur via de novo mutation or from the rise in frequency of standing genetic variants, and will depend on the interplay between many factors including population size, gene flow, and allelic dominance of the causative variants (Nuismer et al., 2012). The repeated evolution of blanched coloration in two independent populations and the young geological age of both dune systems makes it tempting to suggest that blanched alleles may be segregating at low frequency as standing genetic variation in dark soil populations. This could explain a rapid rise in frequency when modulated by selection and/or demographic changes (Barrett & Schluter, 2008; Colosimo et al., 2005). However, additional genome‐wide studies across geological replicates will be necessary to identify specific adaptive alleles and their likely history.

Finally, it is interesting to note that the SBD *H. maculata* population has a much lower census population size than that at WS (based on habitat size and field observations). Thus, it will be important to determine how the blanched phenotype arose and is maintained given that the effects of genetic drift – and the swamping effects of gene flow from dark soils populations – would be amplified in this small population. Therefore, the SBD system provides an interesting demographic counterpoint to the White Sands for studies of the dynamics of small locally‐adapted populations and studies of the genetic architecture of repeated evolution.

### 3.2 Salt Basin dunes and White Sands lizards differ in limb length and possibly habitat use

Dorsal coloration was highly convergent between both populations (Figure 1, Figure 3), with no statical difference in level of blanching (Hedge's g and 95 percent confidence interval for: WS males vs SBD males = 0.21 (0.207, 0.212) – small effect size; WS females vs SBD females = −0.09 (−0.089, 0.085) – negligible effect size).

**FIGURE 4:**
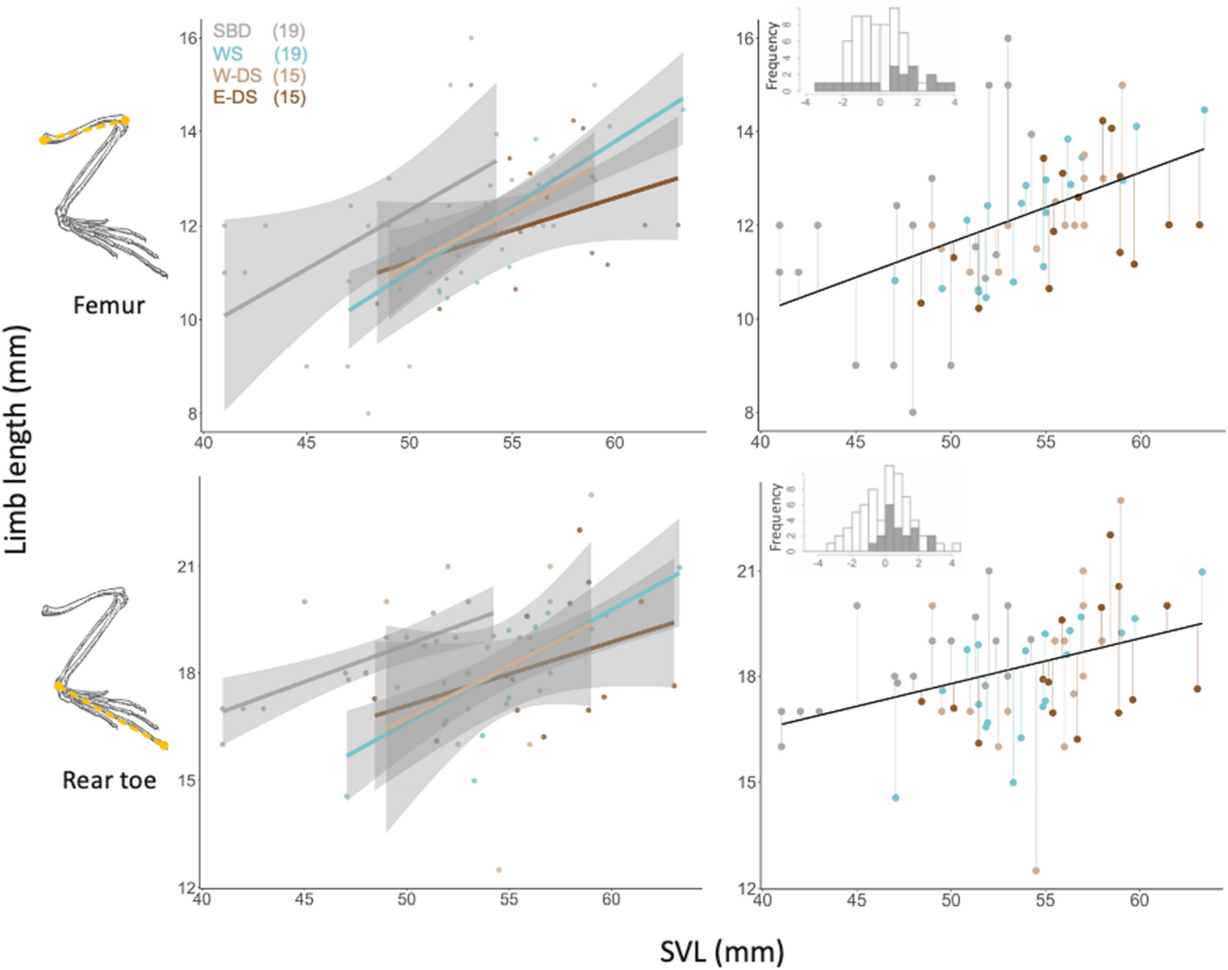
Comparative limb morphometrics across habitats. Salt Basin Dunes lizards tend to have overall longer limbs, assessed by the relationship between Body size (SVL) and femur length (first line), and rear toe length (second line). Numbers in brackets refer to number of individuals analyzed per habitat. Left column: Colored lines represent linear model per habitat, with shading as the 95% confidence interval (gypsum dunes: gray for SBD and blue for WS; dark soils: light brown for West and dark brown for East). Right column: Predicted model and residual distribution similarly color coded by habitat. Points above the correlation line correspond to individuals with limbs longer than expected based on the prediction of relationship between limb and body size (higher residuals). Histograms visualize the residuals distribution with SBD data highlighted in gray. Limb drawings adapted from (Cox & Tanner, 1977).

In parallel with convergence of blanched coloration between SBD and WS *H. maculata,* we also find evidence of divergence in other traits. Limb size showed interesting trends, with SBD *H. maculata* having longer limbs than both dark soil and WS populations (Figure 4), and a more pronounced difference in rear toe length (*p‐value* = .00392) versus femur length (*p‐value* = .0802). Blanched lizards from the WS are usually bigger than their dark soil counterparts (body size and weight), and seem to have longer hindlimbs too; but while rear toe scales with SVL, it does not differ between WS and West dark soil populations (Des Roches et al., 2014).

**FIGURE 5:**
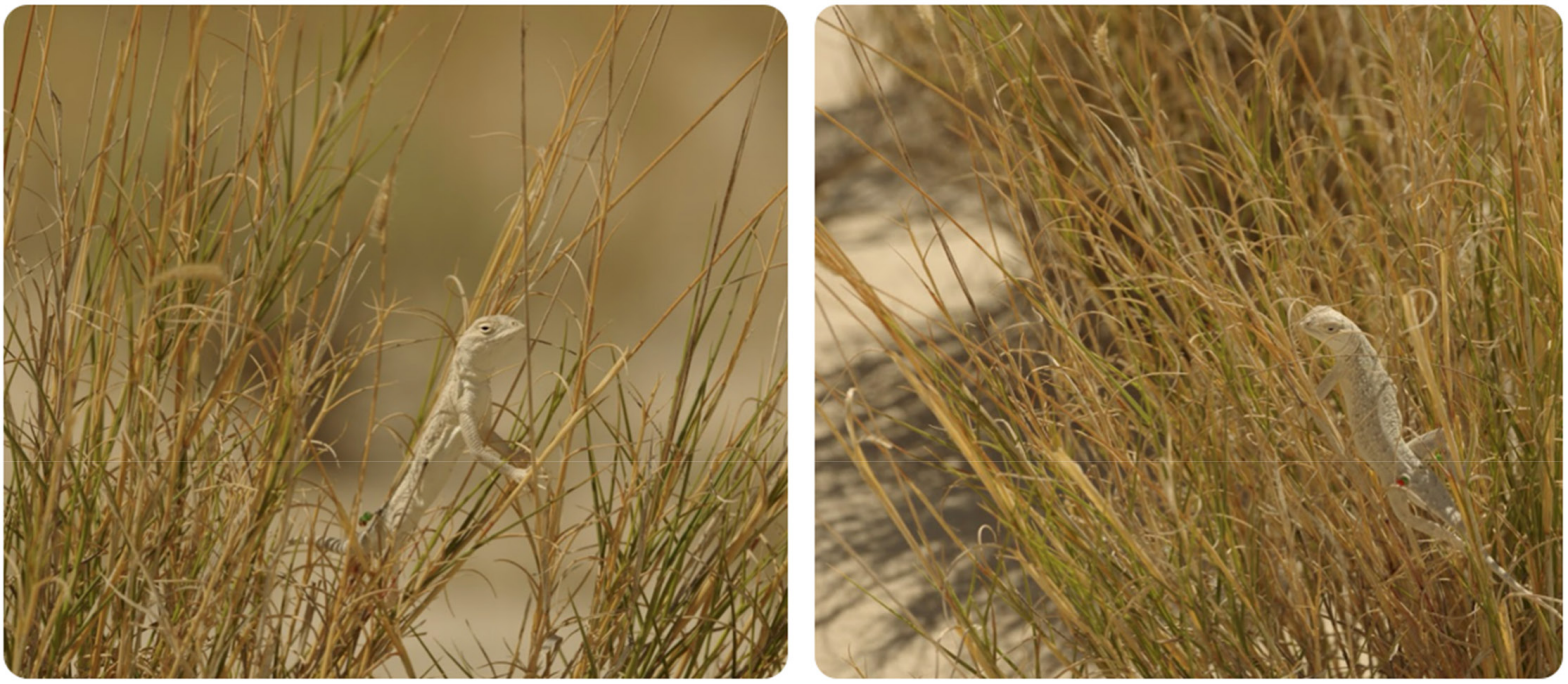
Blanched *H. maculata* exhibiting typical perching behavior, at the Salt Basin dunes. The newly discovered population frequently exhibits a vegetation climbing and perching behavior very rarely recorded for the WS conspecific population. It is possible that the behavior is associated with limb size divergence and absence of perching competitors, but this hypothesis requires further testing. Photo credits: DED.

Because both SBD and WS populations are arenicolous, facing similar locomotion constraints, we might expect their limb lengths to be similar. However, a behavioral observation might be linked to this phenotypical trend: out of 25 SBD lizards captured in the summer of 2018, three (12%) were captured while exhibiting perching behavior. This is a common find in the SBD (TGL and DED, pers. observation) with lizards frequently spotted on branches of sagebrush (*Artemesia filifolia*), trunks of yuccas, and on grass stems (Figure 5). Despite a similar plant community being available, this behavior has very rarely been observed in the White Sands *H. maculata* population (oral communication from 6 researchers, ~20 years of cumulative observations).

Perch use is a key component of the ecological niche of sit and wait foragers (Hager, 2001a), and behavioral shifts in perch use can play a crucial role during adaptation to new environments that differ in predator composition (Losos et al., 2004) or inter vs intra‐specific competition dynamics (Losos et al., 1993). In the WS, where *H. maculata* is not known to perch frequently, its phrynosomatid relative *Sceloporus cowlesi* makes regular use of vegetative perching habitat. At WS, *S. cowlesi* have longer rear toes (Des Roches et al., 2014) and a broader climbing niche (Des Roches et al., 2011) than their dark soils conspecifics. Hind‐limb length is known to be correlated with perch characteristics in other species of arboreal lizards (Losos, 1994; Losos et al., 1997), with a significant contribution of phenotypic plasticity (hatchlings reared on broad perches had longer hind limbs, Losos et al., 2000). Thus, it is tempting to speculate that the absence of a species like *S. cowlesi* in the SBD could allow *H. maculata* to expand their habitat use. Furthermore, the presence of predatory leopard lizards (*Gambelia wislizenii*) in the Salt Basin Dunes, at the ground level, might contribute to selective benefits for periscoping behavior while perching on taller grasses.

We found no significant patterns of head shape divergence in our analyses. Head shape is often correlated with bite force (Anderson et al., 2008) and burrowing movement in sandy habitats (Arnold, 1995). Thus, differences in head size or shape across populations can reflect selection on locomotion on sandy substrate (Bergmann & Berry, 2021); and/or shifts in dietary niche (Herrel et al., 2001; Verwaijen et al., 2002). While WS *H. maculata* tend to have larger heads relative to their West dark soil conspecifics (Rosenblum & Harmon, 2011), we find no evidence of SBD diverging from dark soils nor converging with WS populations for this trait (Figure S3, in appendix S2).

In sum, limb and head morphology are known to impact behavior, locomotion, and habitat use across numerous squamate species. However, the strength of these correlations and the trade‐offs between traits are quite often population or species specific. Thus, focused ecomorphology studies will be needed for SBD *H. maculata* to understand trait heritability and potential links between morphology, behavior, and habitat use in this novel environment.

### 3.3 Divergence within convergence: Adding new power to a classic evolution study system

Overall, we find evidence of both convergence and divergence between the blanched populations of *H. maculata* of the White Sands and the Salt Basin Dunes. Even when convergent morphologies evolve across independent populations in similar habitats, evolutionary patterns are still influenced by population‐level differences in ecology, demography and plasticity (Bolnick et al., 2018). Rarely does a natural system allow researchers to disentangle population‐specific eco‐evolutionary effects, which is why examples of parallel evolution within and among species, in the wild, are so valuable (Arendt & Reznick, 2008; Conte et al., 2012). The WS and SBD systems offer an opportunity to study trait‐specific convergence in species that have adapted to geologically recent habitat shifts (Kocurek et al., 2007; Langford, 2003). Continued comparisons across these systems can shed light on the genetic architecture of repeated evolution in novel, and geologically young, ecological contexts and how natural selection and population demography interact during repeated adaptation in the wild.

## AUTHOR CONTRIBUTIONS

**Telma G. Laurentino:** Conceptualization (lead); data curation (lead); formal analysis (lead); funding acquisition (supporting); investigation (lead); methodology (lead); visualization (lead); writing – original draft (lead); writing – review and editing (lead). **Drew E. Dittmer:** Conceptualization (supporting); funding acquisition (supporting); investigation (supporting); methodology (supporting); writing – review and editing (supporting). **Maggie R. Grundler:** Methodology (supporting); resources (supporting); writing – review and editing (supporting). **Francisco Pina‐Martins:** Data curation (supporting); formal analysis (supporting); investigation (supporting); software (lead); visualization (supporting); writing – review and editing (supporting). **Janey Haddock:** Formal analysis (supporting); methodology (supporting); software (supporting). **Toby J. Hibbitts:** Data curation (supporting); investigation (supporting); resources (supporting); writing – review and editing (supporting). **Erica Bree Rosenblum:** Conceptualization (lead); funding acquisition (lead); investigation (supporting); methodology (supporting); project administration (lead); resources (lead); writing – original draft (equal); writing – review and editing (equal).

## FUNDING INFORMATION

Funding was provided by The Western National Parks Association and SnakeDays (DED); the National Science Foundation (grant DEB‐1754125 to EBR); and the Swiss National Science Foundation (grant P2BSP3_195698 to TGL).

## Supporting information


Appendix S1

Appendix S2

## Data Availability

All used and newly generated phenotypic and genetic data are fully and publicly accessible (Appendix S1). New sequences are publicly availabe at NCBI (individual assession numbers provided in Appendix S1). All phylogenetic inference code and raw sequencing data are publicly available at https://gitlab.com/StuntsPT/the‐colours‐of‐guadalupe.
