# Supplementary material for: Repeated evolution of blanched coloration in a lizard across independent white‐sand habitats
Source: Ecol Evol. 2022 Dec 7;12(12):e9555. doi: 10.1002/ece3.9555 (PMC9729009; doi:10.1002/ece3.9555)
Supplement: Supplementary file 2 — Appendix S2 [file ECE3-12-e9555-s001.docx]

**
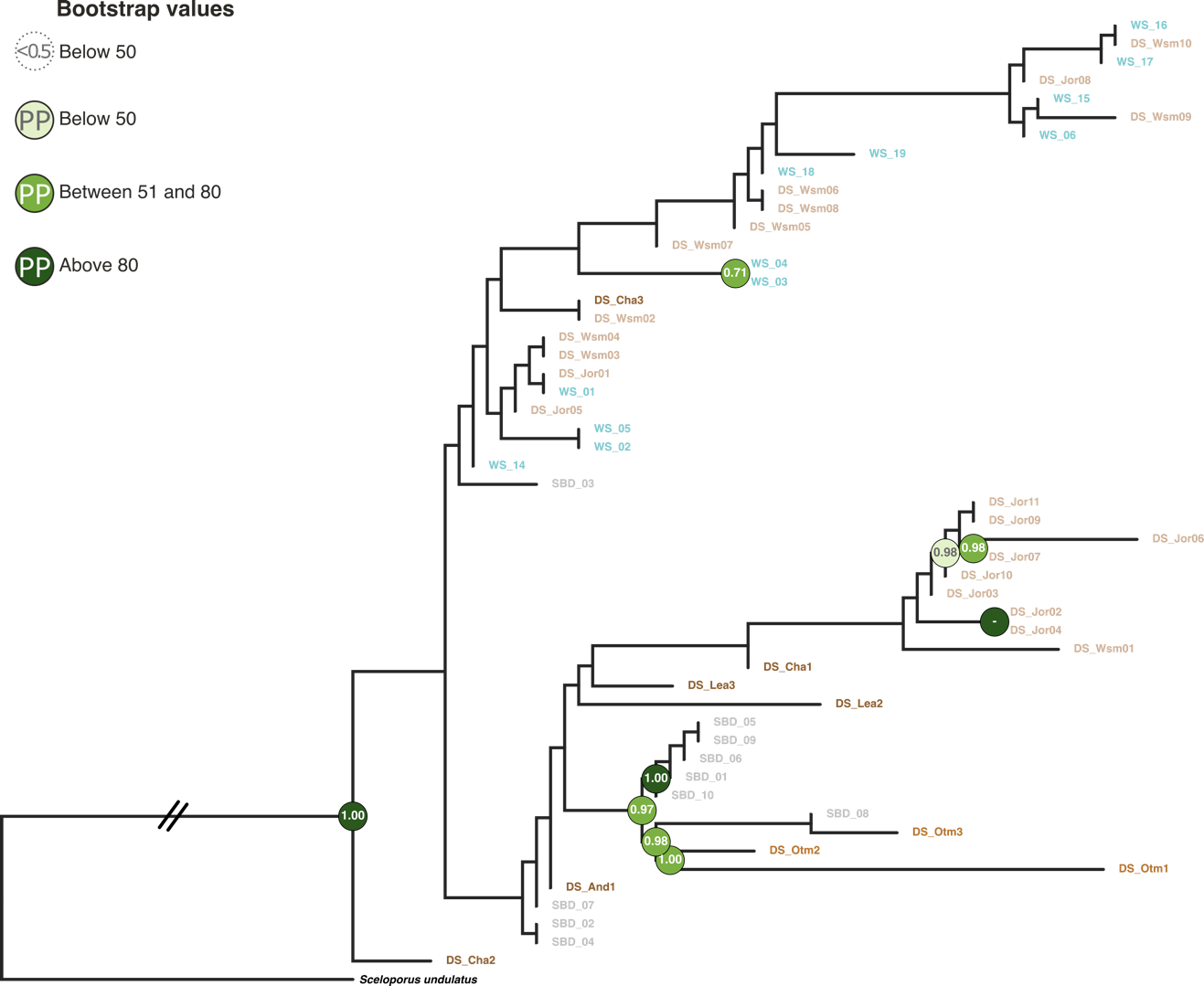
**

**Figure S1 – Phylograms for the mitochondrial gene (ND4, *Top*) and for the melanocortin 1 receptor gene (*Mc1r*, *bottom*)**

Circles at nodes contain information on node bootstrap value (color-coded in green scale) and posterior probabilities (number within). When the boostrap value is below 50 and the posterior probability is below 0.5, a circle is not displayed. Labels for individuals are color-coded by environment and named by population as in Fig.1. Corresponding cladograms in Fig.2.

**
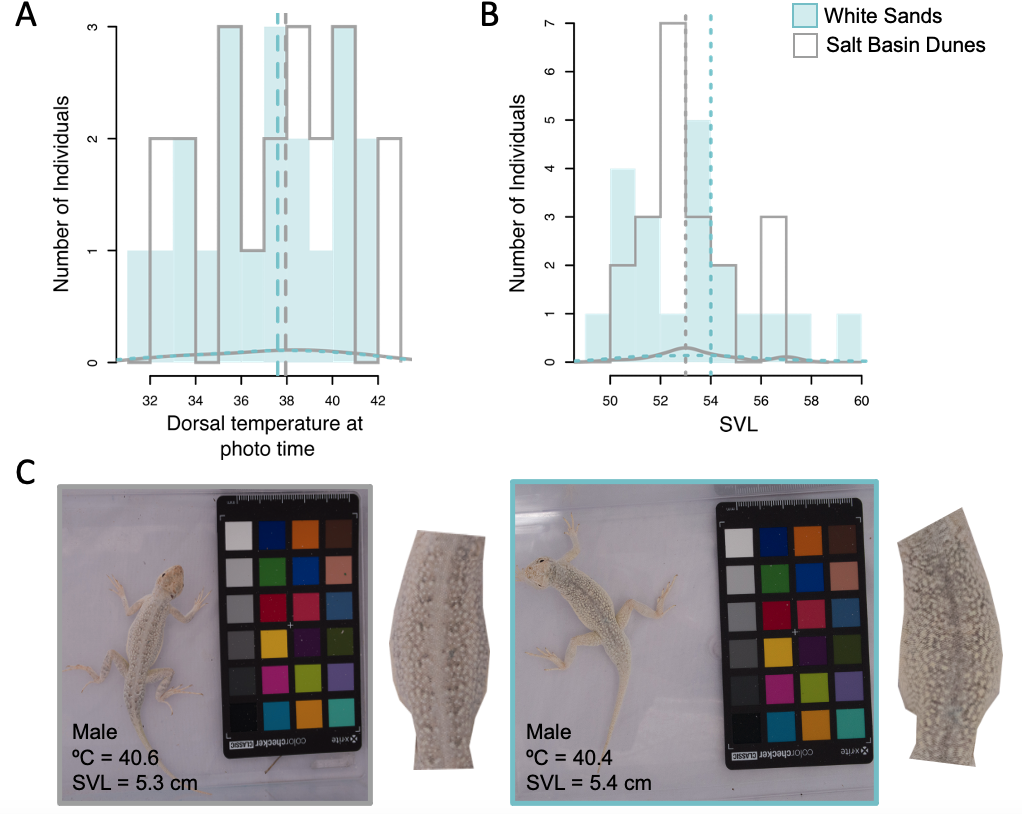
Figure S2 Color analysis workflow**

The samples were paired for sex, dorsal temperature at time of the photo (A) and body size (SVL; B) to avoid confounding effects. Histograms depict the distribution of the dorsal temperatures at the time of photography (A) and SVL (B) for both population samples (White Sands (WS) in filled blue, Salt Basin Dunes (SBD) in grey contour) with overlapping density lines (WS in dashed blue; SBD in grey). Vertical dashed lines mark the median of each population (Temperature: WS = 37.6, SBD = 37.95; SVL: WS = 54, SBD = 53)**.** Color analysis is then focuses on the dorsal area delimited by the transversal lines connecting limb insertions, exemplified by a male pair (**C**; right SBD, left WS).

**
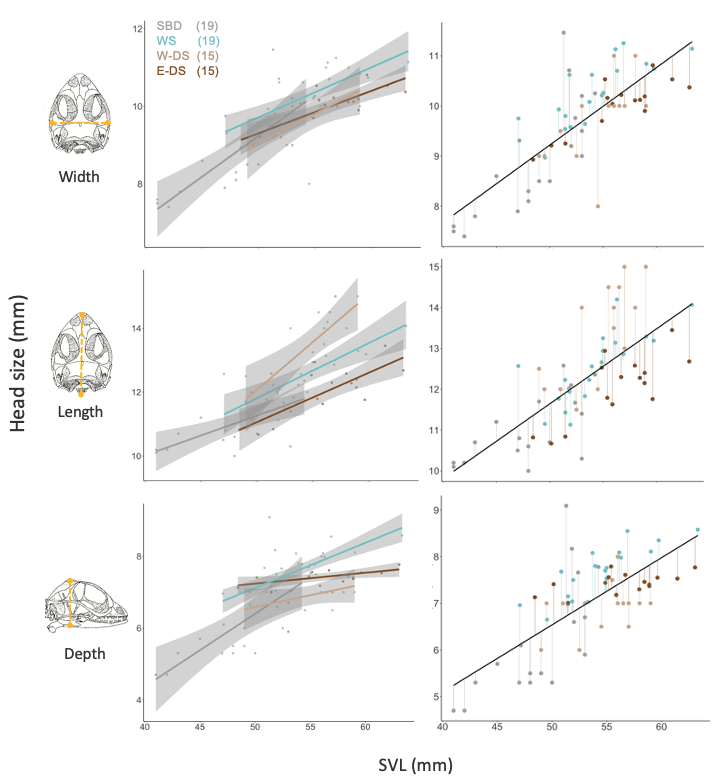
Figure S3 – Comparative head morphology across habitats**

Lizards do not seem to diverge in head size across habitats. Numbers in brackets refer to number of individuals analyzed per habitat. **Left column**: Colored lines represent linear model per habitat, with shading as the 95% confidence interval (gypsum dunes: grey for SBD and blue for WS; dark soils: light brown for West and dark brown for East). **Right column**: Predicted model and residual distribution similarly color coded by habitat. Drawings adapted from (Cox & Tanner, 1977)**.**
